# An Ambulatory Blood Pressure Monitor Mobile Health System for Early Warning for Stroke Risk: Longitudinal Observational Study

**DOI:** 10.2196/14926

**Published:** 2019-10-30

**Authors:** Guangyu Wang, Silu Zhou, Shahbaz Rezaei, Xin Liu, Anpeng Huang

**Affiliations:** 1 Department of Computer Science and Technology Tsinghua University Beijing China; 2 National Institute of Health Data Science Peking University Beijing China; 3 Computer Science Department University of California Davis, CA United States

**Keywords:** ambulatory blood pressure monitor, mHealth, stroke-risk early warning, abnormal blood pressure data analyzing, longitudinal observational study

## Abstract

**Background:**

Stroke, as a leading cause of death around the globe, has become a heavy burden on our society. Studies show that stroke can be predicted and prevented if a person’s blood pressure (BP) status is appropriately monitored via an ambulatory blood pressure monitor (ABPM) system. However, currently there exists no efficient and user-friendly ABPM system to provide early warning for stroke risk in real-time. Moreover, most existing ABPM devices measure BP during the deflation of the cuff, which fails to reflect blood pressure accurately.

**Objective:**

In this study, we sought to develop a new ABPM mobile health (mHealth) system that was capable of monitoring blood pressure during inflation and could detect early stroke-risk signals in real-time.

**Methods:**

We designed an ABPM mHealth system that is based on mobile network infrastructure and mobile apps. The proposed system contains two major parts: a new ABPM device in which an inflation-type BP measurement algorithm is embedded, and an abnormal blood pressure data analysis algorithm for stroke-risk prediction services at our health data service center. For evaluation, the ABPM device was first tested using simulated signals and compared with the gold standard of a mercury sphygmomanometer. Then, the performance of our proposed mHealth system was evaluated in an observational study.

**Results:**

The results are presented in two main parts: the device test and the longitudinal observational studies of the presented system. The average measurement error of the new ABPM device with the inflation-type algorithm was less than 0.55 mmHg compared to a reference device using simulated signals. Moreover, the results of correlation coefficients and agreement analyses show that there is a strong linear correlation between our device and the standard mercury sphygmomanometer. In the case of the system observational study, we collected a data set with 88 features, including real-time data, user information, and user records. Our abnormal blood pressure data analysis algorithm achieved the best performance, with an area under the curve of 0.904 for the low risk level, 0.756 for the caution risk level, and 0.912 for the high-risk level. Our system enables a patient to be aware of their risk in real-time, which improves medication adherence with risk self-management.

**Conclusions:**

To our knowledge, this device is the first ABPM device that measures blood pressure during the inflation process and has obtained a government medical license. Device tests and longitudinal observational studies were conducted in Peking University hospitals, and they showed the device’s high accuracy for BP measurements, its efficiency in detecting early signs of stroke, and its efficiency at providing an early warning for stroke risk.

## Introduction

As a common cerebrovascular disease caused by a hemorrhage or ischemia, stroke has become the primary cause of adult disability, and its incidence is increasing steadily as society ages [1,2]. According to the World Health Organization (WHO), worldwide 1/6 people has a stroke during their lifetime, among which more than one-third of those affected die from their stroke [3]. Worldwide, mortality from stroke and related cardiovascular diseases constitutes around 20% of all deaths, and around 33% of all stroke survivors are affected by depression after [4,5]. It has been estimated that stroke cost in the first half of the 21st century is around USD $1.52 trillion, which is three times as high as the US annual gross tax revenue [6].

To address these challenges, a lot of recent research has focused on stroke prevention. Many risk factors have been shown to be crucial for stroke, including age, gender, hypertension, diabetes, arrhythmia, cholesterol, smoking, and drinking, among others, with hypertension being the most significant controllable factor [7]. In addition, a growing number of studies have demonstrated the predictive value of ambulatory blood pressure monitoring (ABPM) in assessing the risk of first-ever and recurrent stroke [8-10]. According to the American Stroke Association, 77% of patients have a blood pressure (BP) ≥140/90 mmHg when they have a stroke for the first time [11]. Studies have shown that there is a high correlation between Morning Blood Pressure Surge and stroke. As reported by Argentino et al [12], the average probability of stroke incidence from 6 AM to 12 AM is 3.8 times greater than the other times of the day [13]. Furthermore, with each 10-mmHg increase in Morning Blood Pressure Surge, stroke probabilities increase by 44% (*P*=.004) [14]. As a result, a real-time Morning Blood Pressure Surge monitoring system for stroke risk warnings can dramatically reduce stroke probability.

With the rapid development of wireless Internet technology and the increasing number of mobile phone users, mobile health (mHealth) technology has emerged as a potential solution to health care delivery for people with chronic diseases [15-17]. Altintas et al proposed a wearable, 24-hour, low-stress blood pressure monitor system to conduct less painful and less stressful, 24-hour blood pressure monitoring [18]. Yang et al designed a set of General Packet Radio Service ambulatory blood pressure monitoring systems, which consist of a data acquisition module, main control module, communication module, cloud platform, and intelligent terminal [19]. Tsoi et al constructed an integrative electronic health platform with a BP telemonitoring cloud system in elderly community centers to control hypertension [20]. In a recent study, Wu et al introduced the design and development of an mHealth system for strengthening secondary prevention of stroke in rural China and further evaluated its feasibility through a survey of the dominant user group [21]. Moreover, other BP monitoring systems [22-25] use a mixture of solutions, including a homemade blood pressure measurement device, a smartphone- or PC-based management unit, or Bluetooth or ZigBee for data transmission, which help users to measure and manage their daily blood pressure.

However, these studies utilized BP devices based on deflation-type instead of inflation-type BP measurement algorithms, which do not accurately reflect the real state of blood pressure. In clinical diagnosis, most pathological changes inside blood vessels may not be captured by deflation-type BP measurement [26,27] because the mechanical behavior of the brachial artery cannot be restored to a normal state within a sharp transition from inflation to deflation. In addition, the abnormal signals generated from Morning Blood Pressure Surge monitoring of the existing approaches do not provide early-risk alert of a stroke to patients or relatives in real-time. Driven by this motivation, we designed a new ABPM mHealth system which can prevent a stroke in real-time. This system consists of two major parts: a new ABPM device for BP monitoring and data collection, and an abnormal BP data analysis algorithm at a data center for real-time early risk warning services. Significantly, an inflation-type blood pressure measurement algorithm has been proposed and embedded into the new device, which helps to avoid the elastic deformation of blood vessels and any interference signals during measurement. At the data center, the abnormal BP data analysis algorithm runs to classify stroke-risk levels based on real-time BP data, medical records, and health state information. To evaluate the ABPM mHealth system, a longitudinal observational study was conducted, which demonstrated the accuracy and usefulness of the early stroke-risk warning function.

## Methods

### Ambulatory Blood Pressure Monitor Mobile Health System and Architecture

**Figure 1:**
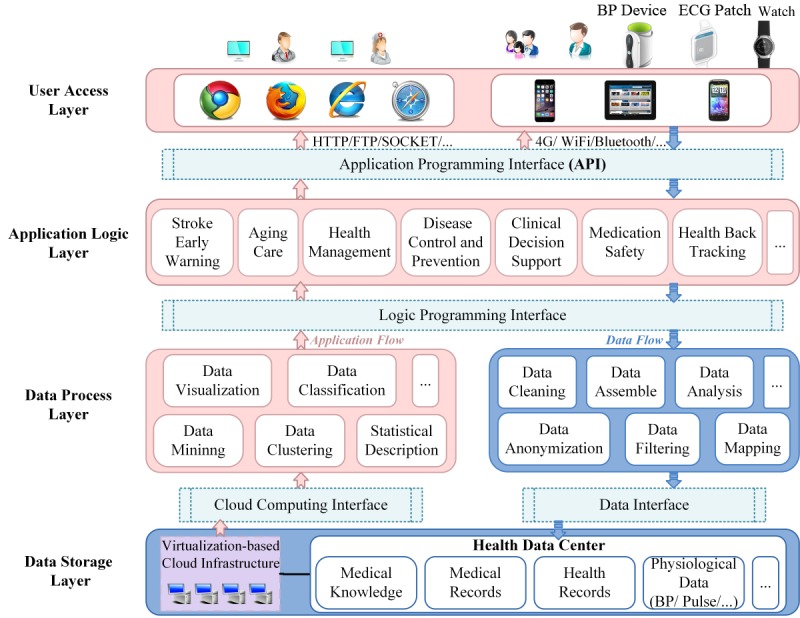
The four-layer ambulatory blood pressure monitor mobile health architecture for stroke-risk early warning. BP: blood pressure; ECG: electrocardiogram.

To enable the ABPM mHealth system to warn of early risk of stroke, a four-layer functional architecture was developed (Figure 1). These layers included the user access layer, the application logic layer, the data process layer and the data storage layer, the structure and function of which are described below.

#### User Access Layer

This layer provides the interfaces between the users/devices and the data center. In this layer, an ABPM device is used to collect data from potential stroke patients through online and application interfaces that are available to users, as shown in Figure 1. However, admission authority and transmission issues should be considered during this part of the process, as user password and text verification codes are used for identification, and Cross-Site Request Forgery defense and transport layer security are applied to secure information delivery.

#### Application Logic Layer

This layer involves multiple forms of business intelligence logic, such as health state maintenance, clinical decision support and treatment, and nursing and care. Among them, this paper focuses on an abnormal BP data analysis algorithm running in the data center for stroke early warning.

#### Data Process Layer

In this layer, all data are processed to remove electronic noise and motion interference. Then, the preprocessed signals are analyzed, mined, and visualized for the purpose of stroke early-risk warning.

#### Data Storage Layer

In this layer, it is necessary to handle missing or damaged data to guarantee data integrity.

In this study, a new ABPM mHealth architecture was developed to provide early stroke warning in real-time. Here, we focus on two major parts: at the frontier for real-time data collection, the device embedded with an inflation-type BP measurement algorithm, and at the data center, an abnormal BP data analysis algorithm for real-time early stroke warning using users’ terminal interfaces.

### At the Frontier: An Ambulatory Blood Pressure Monitor Device for Inflation-Type Blood Pressure Measurement

**Figure 2:**
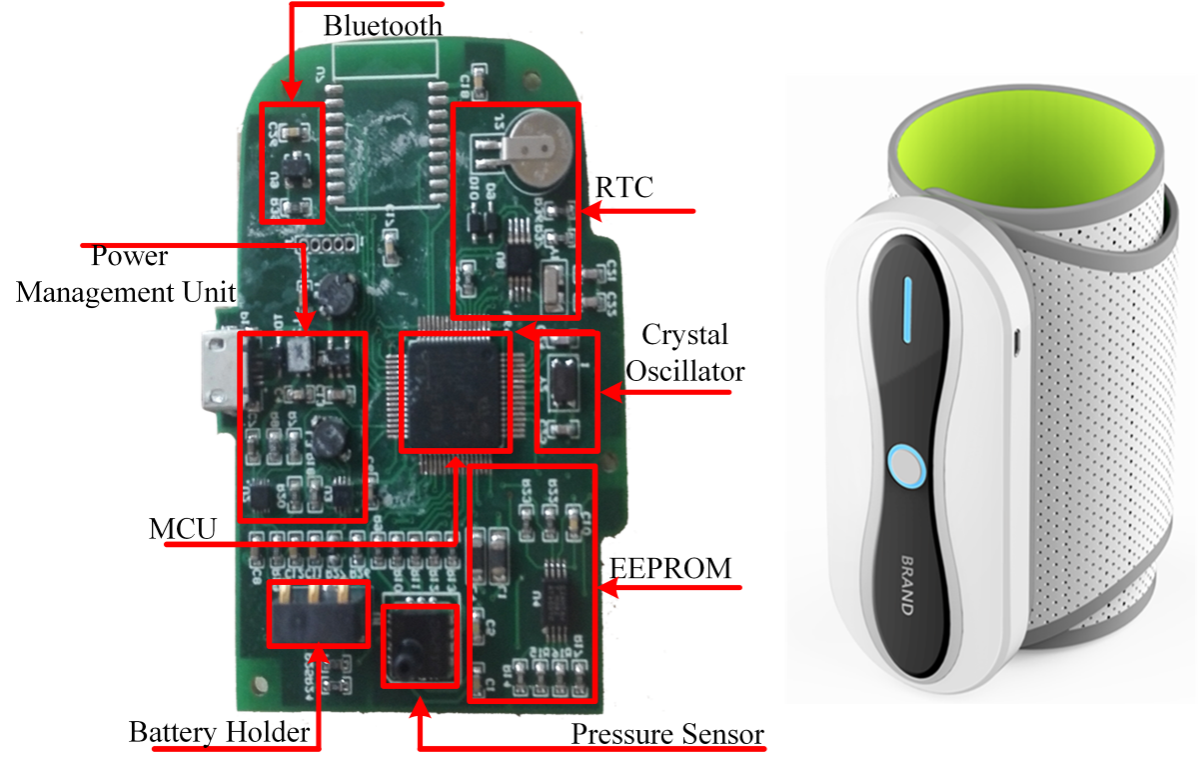
Printed circuit board display of the new ambulatory blood pressure monitor device. RTC: real-time clock; MCU: microcontroller unit; EEPROM: electrically erasable programmable read-only memory.

As previously mentioned, most pathological changes inside blood vessels may not be captured properly by BP devices with a deflation-type measurement. To solve this issue, our device is instead embedded with an inflation-type BP measurement. For different stroke patients, this device can be adapted to physiological and external factors as well. Additionally, our device can be automatically pumped to the appropriate maximum inflation pressure values according to the preset systolic blood pressure, which helps the new device adapt to users’ health conditions for raw pressure data collection. For details about the inflation-type BP Measurement, please see Multimedia Appendix 1. Our developed ABPM device is shown in Figure 2.

There are multiple advantages to this device, including: (1) the adoption of the inflation-type measurement algorithm, which is the first commercial device that can capture vasculopathy information in detail; (2) that it is personalized and user-friendly, it can inflate to a suitable pressure level according to users’ individual physiological states, and it automatically adjusts inflation speed according to users’ activity states (6 mmHg/second while awake, and 4 mmHg/second during sleep), which can be recognized by a 3-axis sensor; and (3) the pump and cuff are bound together to reduce noise, in comparison with existing commercial products.

### Early Warning Services for Stroke Risk at the Data Center

As mentioned above, stroke risk can be controlled if a Morning Blood Pressure Surge monitoring system can generate early warnings for stroke risk in real-time. To achieve this goal, we needed to detect abnormal stroke-related signals, which would then be uploaded to the data center. However, existing research for stroke prevention mainly focuses on statistical studies about public health. Kansadub et al investigated classification algorithms for a national- or group-level, stroke-risk increasing or reducing prediction model, which included methods such as naive Bayes, decision trees, and neural networks [28]. Yang et al constructed a predictive model for stroke based on multiple regression analyses of multiple climate factors [29]. Most of these studies focus on stroke risk factor analysis, not a real-time solution of abnormal stroke signal analysis.

In this study, an abnormal BP data analysis algorithm (see Multimedia Appendix 2) is used to provide early warning to patients. The algorithm classifies stroke risk levels as low, caution, or high. In this algorithm, a total of 88 features, including BP real-time data and health state information, are selected for classification, some of which are listed in Table 1 (health state information is from patient records). When this algorithm is running at the data center, all numerical values are normalized to 0 or 1 for empty numeric attributes, or they are normalized to the average value or 0 to replace empty Boolean attributes. This strategy is helpful for handling missing data in the algorithm. All necessary information is fed into the abnormal BP data analysis algorithm to determine the level of stroke risk. In this algorithm, the support vector machine approach is used to train data and simulated annealing is adopted to tune the hyper-parameters. Additionally, a 10-fold cross-validation was applied to refine the hyper-parameters.

**Table 1:** Input data and features (partial).

Feature, characteristic		Comments
**24-hour ABPM^a^**		
	Hypertension grading	Calculated by WHO^b^ hypertension definition
	BP^c^ load	Percentage of elevated pressure above a defined threshold
	Dipper	Whether or not blood pressure falls at night compared to daytime values
	Morning surge	Morning SBP^d^/preawake SBP
	SBP, max	The highest SBP in a given time window
	DBP^e^, max	The highest DBP in a given time window
	SBP, min	The lowest SBP in a given time window
	DBP, min	The lowest DBP in a given time window
	SBP, mean	Average SBP in a given time window
	DBP, mean	Average DBP in a given time window
	PP^f^, mean	Average SBP minus average DBP
**Personal information**		
	Age	Years
	Gender	1=Man, 0=Woman
	Height	Centimeters
	Weight	Kilograms
**Lifestyle**		
	Smoking	1=Yes, 0=No
	Drink	1=Yes, 0=No
	Physical inactivity	Amount of exercise per week
**Patient history**		
	Stroke	1=Yes, 0=No
	Diabetes mellitus	1=Yes, 0=No
	High blood cholesterol	1=Yes, 0=No
**Family history**		
	Stroke	Family members with stroke
	Hypertension	Family members with hypertension

^a^ABPM: ambulatory blood pressure monitor.

^b^WHO: World Health Organization.

^c^BP: blood pressure.

^d^SBP: systolic blood pressure.

^e^DBP: diastolic blood pressure.

^f^PP: pulse pressure.

### Design of Service Interfaces to Enable Stroke-Risk Alerts

**Figure 3:**
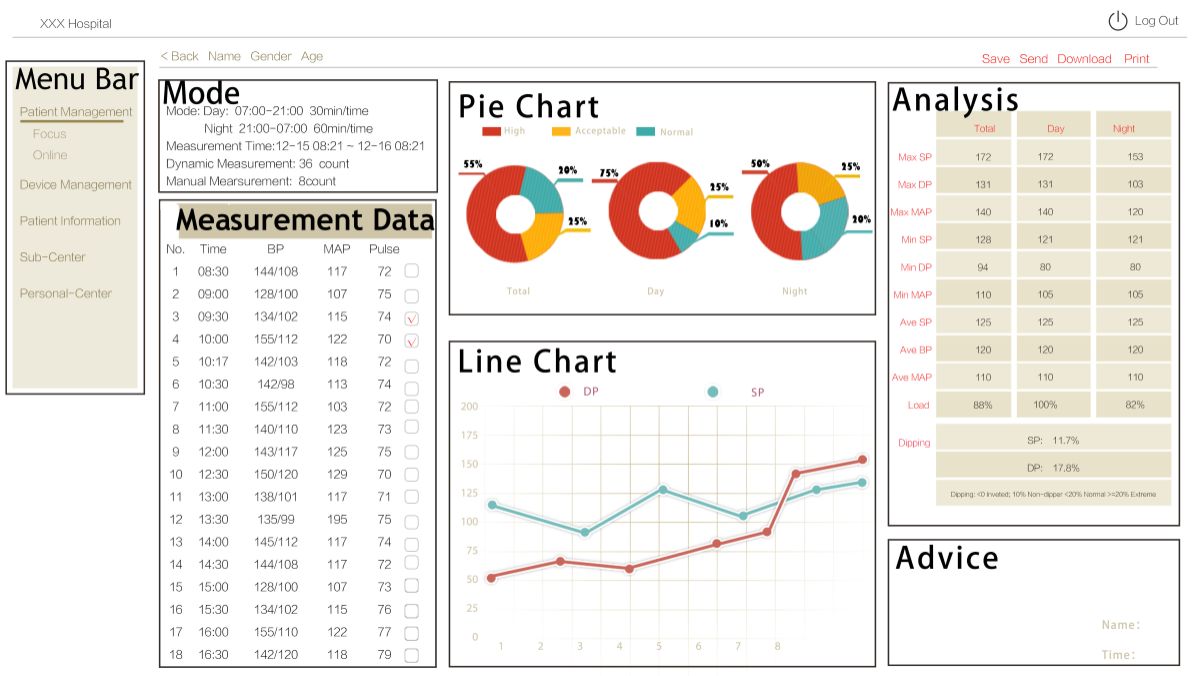
The Web user interface of the Health Data Center.

**Figure 4:**
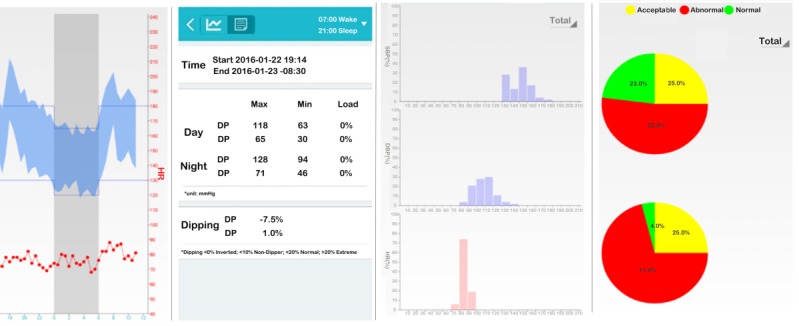
Data analysis and visualization interfaces for app users.

In practice, stroke risk can be reduced if a Morning Blood Pressure Surge monitoring system can generate early risk warnings for stroke in real-time. At the data center, an application programming interface (API) was deployed to provide reliable and stable data transmission between users and the service center. We designed two types of user interfaces: one for doctors to manage and diagnose patients on the website, and the other for data presentation and analysis on users’ phones (Figures 3 and 4).

As shown in Figure 3, a doctor can review the medical signals, give instructions, or print a hard copy of diagnosis results. Users can display data in many forms on their phone, such as with histograms, pie charts, or line charts, as well as a 24-hour ABPM analysis report. As shown in Figure 4, the mobile app on smartphones provides patients with evaluation and visualization services, which are important for health management and stroke risk warning. This app has a robust network connection by using several different operations, including calling a local database instead of logging into the server again, providing rollback database operations to avoid inconsistency, using hash values to avoid repeated writing, etc.

### Evaluation of the Ambulatory Blood Pressure Monitor Mobile Health System

Through a longitudinal observational study, our new inflation-based ABPM device and our stroke-risk early warning system were evaluated and compared against the gold standard mercury sphygmomanometer. All studies were carried out in the community hospitals of Peking University.

Firstly, to calibrate our device, we used the ProSim 8 simulator (FLUKE Corporation, Everett, Washington, United States) to generate a series of source-simulated signals (pulse/sleevelet waves) for calibrating the accuracy. In this study, the HEM-7207 electronic sphygmomanometer (Omron Corporation, Osaka, Japan) was used as a reference as it is in common use. The simulation data sets contain 20 variables: 7 standard BPs, 7 patient conditions, 3 arrhythmias, and 3 respiratory types related to BP. The corresponding systolic blood pressure (SBP), diastolic blood pressure (DBP), and heart rate ranges in the data set are 60-255 mmHg, 30-195 mmHg, and 40-180 times/minute, respectively (see Multimedia Appendix 1 for the results of our device tests).

Secondly, we further conducted device tests according to a gold standard for agreement analysis. According to the regulations from the China Food and Drug Administration which have been in place since 2015, a new BP device can be approved if tests are passed by using simulation signals [30]. This is the reason that simulation signals for device testing are enough to verify a BP device, without the need for real-world clinical trials. In this study we still performed 100 participant tests in Peking University community hospitals, and they were used for correlation coefficient and agreement analysis. A total of 120 participants were involved in these tests, including 56 females and 64 males. The mean age of participants was 49.5 years old (SD 15.6; range 22-86 years old), the mean weight was 68.1 kilograms (SD 19.7; range 45-103 kilograms), the mean height was 166 centimeters (SD 10.7; range 151-187 centimeters), and the average heart rate was 70.6 beats per minutes (SD 15.4; range 45-125 beats per minute). For each subject, BP was measured by the gold standard device, the mercury sphygmomanometer.

Finally, to validate the risk prediction function of this system, 209 patients were chosen as participants in Peking University community hospitals from April 12, 2017, to December 13, 2018. These subjects were required to use our app to upload personal health state information and wore the ABPM devices 24 hours a day to collect real-time BP data. Meanwhile, doctors made professional stroke-risk diagnoses of the subjects based on a comprehensive examination, including hematology, electrocardiograms, magnetic resonance angiograms, etc.

## Results

### Device Test Using Simulated Signals

The device test using simulated signals showed that our new device outperformed the HEM-7207 electronic sphygmomanometer in most cases (for more detailed information see Multimedia Appendix 1). According to error analyses of the two devices, in our device the mean error for SBP was 0.55078 mmHg (SD 0.517412 mmHg) and for DBP it was 0.32185 mmHg (SD 0.39169 mmHg). However, the mean error for SBP and DBP in the HEM-7207 are 2.15 mmHg and 1.85 mmHg, respectively. It shows that our device has achieved measurement consistency and repeatability requirements.

### Device Test Using a Standard Mercury Sphygmomanometer

We further conducted device tests for agreement analysis using the gold standard mercury sphygmomanometer. The measurement results showed that in our device the SBP was 125.45 mmHg (SD 25.2 mmHg) and the DBP was 77.92 mmHg (SD 23.5 mmHg), while for the mercury sphygmomanometer the SBP was 126.72 mmHg (SD 24.7 mmHg), and the DBP was 79 mmHg (SD 23.8 mmHg). The SBP difference between our device and the mercury sphygmomanometer was 1.34 (SD 2.95), while the DBP difference was 1.27 (SD 2.77). The correlation coefficient of our device with the mercury sphygmomanometer was 0.958 during the SBP test and 0.912 during the DBP test. This means that there is a high linear correlation between the two measurement approaches. In terms of the difference between the two measuring devices, their mean and standard deviation values are acceptable for practical applications.

**Figure 5:**
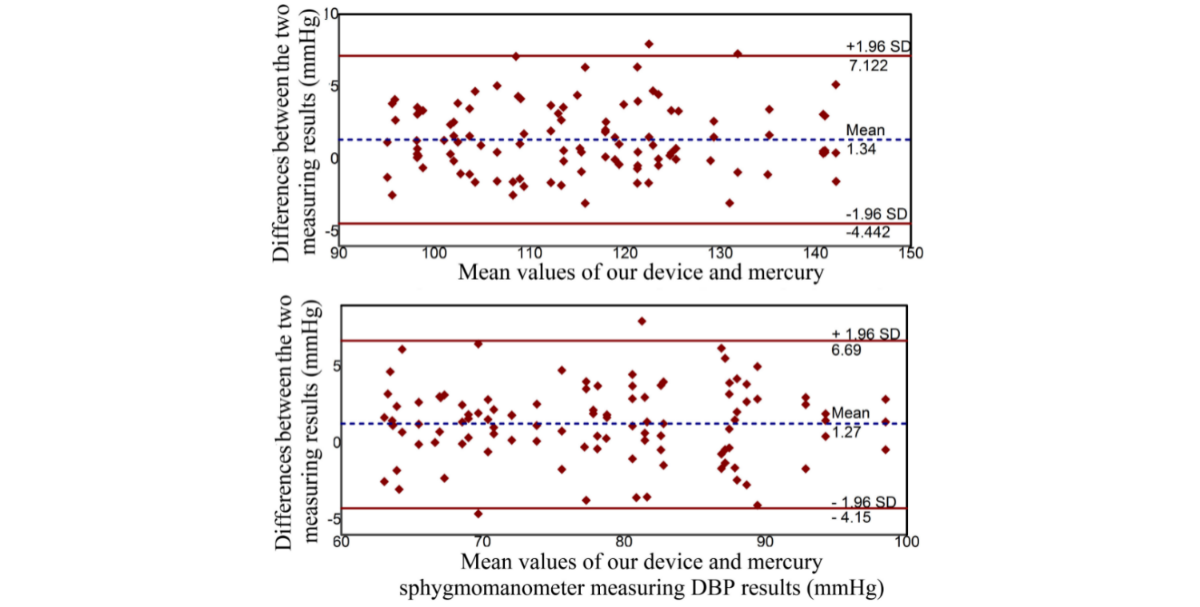
Agreement analysis between our device and the sphygmomanometer using a Bland and Altman plot. DBP: diastolic blood pressure.

As shown in Figure 5, the consistency and agreement of our device with mercury was evaluated. In this figure the Bland and Altman plot [31] was adopted, wherein the X-axis represents the mean of the SBP/DBP measurements of our device and the mercury sphygmomanometer and the Y-axis shows the difference between the two measurement approaches. The results of the correlation coefficient and agreement analysis clearly show that our new device met the clinical requirements and can be used to support clinical diagnosis.

### Longitudinal Observational Studies of the Ambulatory Blood Pressure Monitor System

We randomly selected 20% of subjects out of the 209 participants as a test set, another 20% as a validation set, and the remaining participants were used as a training set. To evaluate the performance of our risk prediction model, we compared our abnormal BP data analysis algorithm with fully connected neural networks and random forests. To evaluate the performance of our proposed methods and the alternatives, F1-score, specificity, accuracy, precision, recall, and area under the curve (AUC) parameters were adopted (Table 2). As shown in Table 2, the proposed abnormal BP data analysis method achieved better performance for the three risk levels. To be specific, for the high-risk level the accuracy was 0.885 and the AUC was 0.912.

**Figure 6:**
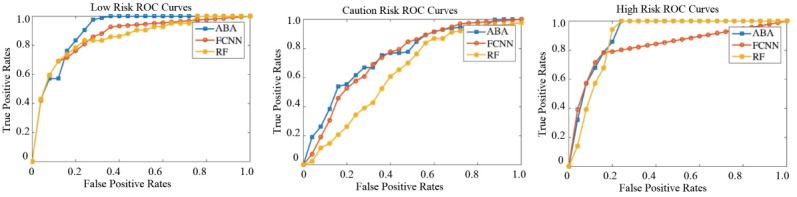
ROC curves of stroke risk prediction. ROC: receiver operating characteristic; ABA: abnormal blood pressure data analysis; FCNN: fully connected neural networks; RF: random forest.

Figure 6 presents the Receiver Operating Characteristic (ROC) curve of three risk levels: low, caution, and high. We can see the ROC curve is smooth, which means that there is no overfitting. Moreover, the abnormal BP data analysis algorithm is closer to the top left corner compared to the other two risk detection models, which means that our system achieved better prediction performance.

**Table 2:** Test performance of the abnormal blood pressure data analysis algorithm compared to other models.

Risk levels, models		F1-score	Specificity	Accuracy	Precision	Recall	AUC^a^
**Low**							
	FCNN^b^	0.645	0.867	0.835	0.588	0.714	0.863
	RF^c^	0.552	0.702	0.725	0.419	0.809	0.859
	ABA^d^	0.659	0.867	0.840	0.596	0.738	0.904
**Caution**							
	FCNN	0.771	0.628	0.710	0.790	0.753	0.731
	RF	0.571	0.785	0.565	0.794	0.446	0.636
	ABA	0.786	0.629	0.725	0.795	0.776	0.756
**High**							
	FCNN	0.528	0.936	0.875	0.560	0.500	0.827
	RF	0.519	0.848	0.83	0.434	0.646	0.894
	ABA	0.673	0.953	0.885	0.618	0.714	0.912

^a^AUC: area under the curve.

^b^FCNN: fully connected neural networks.

^c^RF: random forest.

^d^ABA: abnormal blood pressure data analysis.

## Discussion

### Principal Results

With the application of mobile and communication technology in the medical field, mHealth, which can provide real-time health monitoring service for the elderly or patients with chronic diseases, has attracted a lot of research attention [32-35]. In this study, we proposed a novel ABPM mHealth system that can facilitate accurate BP measurement and generate early warnings for stroke risk in real-time. This paper describes the ABPM mHealth architecture, the device with its inflation-type BP measurement, and the stroke-risk prediction method at the data center. In the end, we carried out a longitudinal observational study and evaluated the proposed mHealth system.

Using mobile devices such as phones and patient monitoring devices, mHealth supports personal health management and public health activities [36]. Recent research has mainly focused on the technical aspect of research design, such as the Android platform, Internet of Things technology [37], cloud platforms [38], medical information systems, wireless monitoring equipment, Bluetooth technology, wearable devices [39], and so on. However, most of these studies are still in the pattern design stage or focus on how to develop or perfect the existing systems or mHealth applications, paying less attention to the user's demand.

We designed a novel ABPM mHealth system to carry out early warning of stroke risk. To capture and monitor a person’s BP profile and support clinical decision, a certified ABPM device (No. 20172201157) was designed and developed. Our device is customized, adaptive, and user-friendly, as it can pump according to a personal health condition, which allows for a personalized measurement with precision. After that, a risk prediction abnormal BP data analysis method is used to process the BP data, which enables this system to provide early warning of stroke risk to users in real-time.

As previously mentioned, a new inflation-type BP method was proposed in this study, and it was shown to be more effective compared to existing solutions. Currently there are two existing inflation-type BP methods, but neither is adaptive. The first one is the stop-and-wait inflation method [40] that contains several stopping points preset for an inflation process. During inflation, its pump is set to stop and wait for 2-3 seconds at each stopping point. In the 2-3 second waiting period, pressure sensor data is processed and then the algorithm decides whether to stop inflation. This method can reduce motion interference, but blood vessels under long-time compression increase inaccuracy. The second method is nonstop inflation [41] where the highest pulse wave peak is regarded as a criterion to calculate the mean arterial pressure (MAP), and then the MAP is used to determine the inflation-ending condition. Although the method is fast and simple, it is easily affected by motion interference since it introduces jitter signals that may be detected as the highest pulse wave peak. However, the detected highest pulse wave peaks in these cases do not represent a real criterion for MAP calculation. Therefore, the existing methods cannot be used to achieve accurate inflation-type BP measurement. To solve these existing problems, our work uses a sliding time window to optimize inflation where the cuff is linearly pumped by a stable pump algorithm and raw pressure data is collected and processed.

To evaluate the ABPM mHealth system performance, system evaluation and longitudinal observational studies were conducted, and 341 risk warning events were recorded, with these events then used to evaluate system performance in practice. Among these participants, there was a stroke patient who had 2-3 strokes per year over the past two years due to not following doctor instructions. This outpatient wore our device during the study, so their blood pressure was measured every day and medication ended up being taken whenever our system generated the early warning signals of a stroke. As a result, there were no recurrent stroke events for a whole year for this patient. Another important case was an emergency outpatient whose blood pressure was rising abnormally and triggered a warning alert in our system. The patient was sent to a hospital in time after our system notified his doctors and relatives. These cases show that our system can help patients realize their risk in real-time, which can reduce stroke risk and improve users' health conditions.

In addition to that, our system was able to generate periodic notifications and warnings about the patients' conditions. This ambulatory system could encourage users to pay attention to their medication states, diet, and emotional health, among other things, which could improve compliance with risk self-management. As a result, the new ABPM mHealth system equipped with the abnormal BP data analysis algorithm was proven to be a useful tool for controlling and preventing stroke, and it could be applied to detect early risks of cardiovascular disease and other BP-related diseases.

### Limitations

This work is interdisciplinary in nature. Thus, we cannot cover all the details about the new ABPM mHealth system in one paper. This paper focused on how to construct an ABPM mHealth system for early warning of stroke risk. In terms of this system, the frontier (an inflation-type BP measurement device) and the data center (an abnormal BP data analysis algorithm for early risk alert) are highlighted in detail. The mHealth working principles are omitted because they follow standard practice right now.

To the best of our knowledge, this new ABPM device is the first commercial product that measures blood pressure during inflation. To improve measurement accuracy, a sliding window strategy that can improve inflation-type measurement and reduce motion interference substantially was adopted. Moreover, our ABPM device is equipped with a proportional integrative derivative controller responsible for linear inflation. The device has room for further improvement, such as insulation of mechanical noise, miniaturization in terms of size, reliability and stability of data transmission, battery consumption and continuation, multiple parameter processing, etc.

Most significantly, the abnormal BP data analysis algorithm can be used as clinical decision support, with a supervised machine learning algorithm to classify the risk level of a stroke patient in real-time. The algorithm has been implemented in our data center and was tested for performance. The performance of the abnormal BP data analysis algorithm was compared with the random forest algorithm and a fully connected neural network, showing that our abnormal BP data analysis algorithm has better performance in accuracy, precision, and recall. However, there remains work left for future studies, such as the development of an algorithm without the need for decision support from a remote data center, how to offer privacy protection, and so on.

In this study, we recruited 209 patients as longitudinal observational subjects in the community, which is not a big number in terms of clinical trials. This limited the training of a more accurate stroke early warning model and the validation of the performance of the ABPM mHealth system. We plan to carry out the study with a more comprehensive experimental design to test our ABPM mHealth system.

## Appendix

Multimedia Appendix 1 The new device with an embedded inflation-type BP measurement algorithm: design and test.

Multimedia Appendix 2 An abnormal BP data analyzing algorithm for stroke-risk early warning.

## Abbreviations

ABPM: ambulatory blood pressure monitor
AUC: area under the curve
BP: blood pressure
DBP: diastolic blood pressure
MAP: mean arterial pressure
mHealth: mobile health
PP: pulse pressure
ROC: receiver operating characteristic
SBP: systolic blood pressure
WHO: World Health Organization
