# Supplementary material for: An Ambulatory Blood Pressure Monitor Mobile Health System for Early Warning for Stroke Risk: Longitudinal Observational Study
Source: JMIR Mhealth Uhealth. 2019 Oct 30;7(10):e14926. doi: 10.2196/14926 (PMC6913731; doi:10.2196/14926)
Supplement: Multimedia Appendix 1 [file mhealth_v7i10e14926_app1.pdf]

Multimedia Appendix 1

The New Device with Embedded Inflation-Type BP Measurement Algorithm: Design and Test.

1. An Inflation-Type BP Measurement Algorithm Design

As well-known, most pathological changes inside blood vessels may not be captured properly by BP devices with a deflation-type BP measurement. To address this issue, we propose an inflation-type BP measurement, as shown in Algorithm 1.

Algorithm 1: Steps of an Inflation-Type BP Measurement Algorithm.

1. Initializing customer variables, such as pump rotation speed, a time-window size, etc.
2. While individual-customized inflation is not ending, take actions below,
   2.1 Run the device pump under the control of stable inflation, and collect users' raw pressure sensor data,
   2.2 Abstract pulse waves from the raw pressure sensor data above,
   2.3 Detect and label critical signals of abstracted pulse waves.
3. Noise and Interference signals are filtered out
4. All involved data are dynamically updated in order to avoid an optimal local trap.
5. Sliding to the second or next time window, and repeating Steps 2 and 3, if the individual-customized inflation ending condition is not matched.
6. Otherwise, all collected data are input into the BP calculation model and output BP results.

In Algorithm 1, a sliding time window approach is deployed to collect raw pressure data, in which the algorithm can adapt to users' health condition in order to improve measurement accuracy. Working principles of this algorithm with the sliding time window are illuminated in Figure 1. Within each time window, a cuff is linearly pumped by a stable pump algorithm for raw pressure data collection with precision. From these raw pressure data, pulse waves are abstracted, and their peaks are labeled as critical signals. Within each time window, abnormal signals are all filtered out.

In Figure 2, an example is shown how the algorithm gets optimal results globally. In the figure, the peak *b* is at the edge of the 21st time window, but it is a start point of a downtrend, and would be easily mistaken as an abnormal signal within its own time window. To avoid it, our algorithm filters out abnormal signals with two or more neighboring time windows, e.g., 21st and 22nd time windows combined together, so as to find that Peak *c*, not Peak *b*, is an abnormal signal.

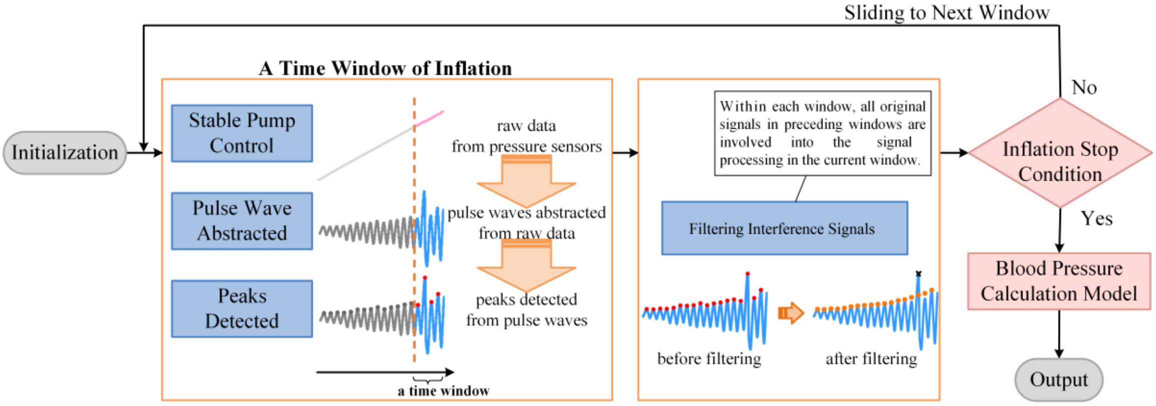

Figure 1. Flowchart of the inflation-type blood pressure measurement mechanism.

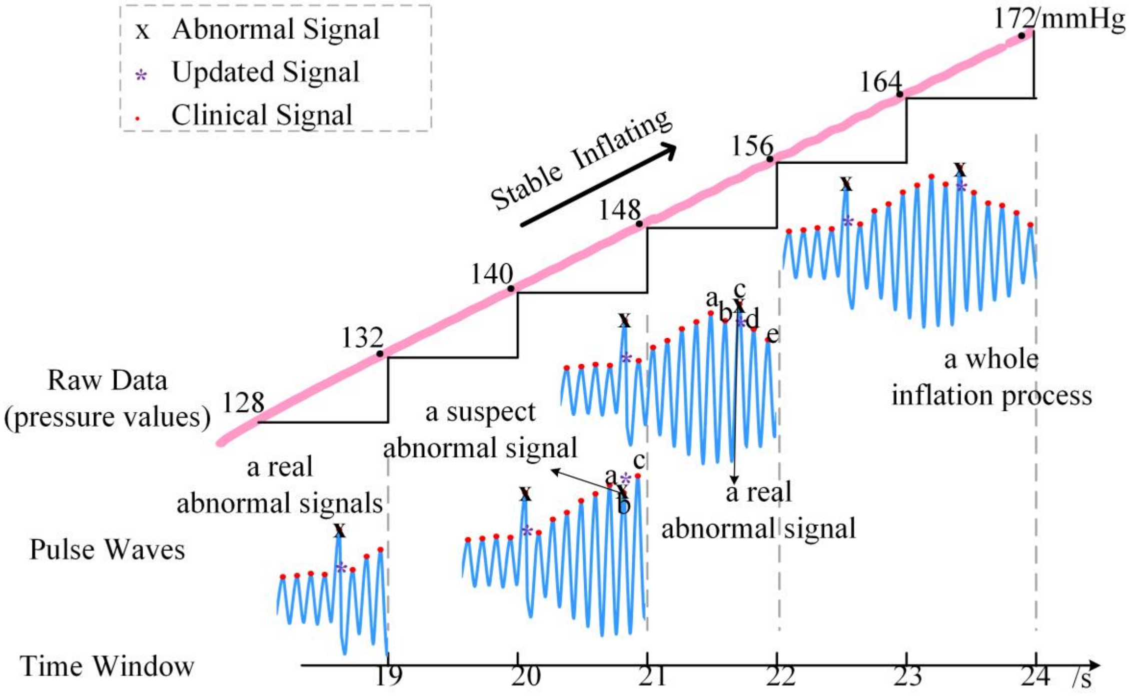

Figure 2. An optimization example of avoiding an optimal local trap in our algorithm.

Additionally, to provide more personalized measurement sets for different stroke patients, this device can be adapt to physiological and external factors as well. Results show that our device can be automatically pumped to the appropriate maximum inflation pressure values according to the preset systolic blood pressure.

2. Stable Control Design of Linear Pump Inflation for this new Device

Most ABPM devices pump the cuff in a non-linear fashion, which introduce more noise and interference signals due to unstable inflation speed. To handle this issue, Proportional-Integral-Derivative (PID) controller is adopted as an inflation stable control mechanism. A PID controller is a control loop feedback mechanism which continuously calculates an error value *e(t)* as the difference between the desired inflation speed *V_0* (6 mmHg in this study) and measured inflation speed. Then, the controller obtains a correction based on its proportional, integral, and derivative terms to get the value *u(t)* of the pump signal, as follows.

$$V = \frac{P_i - P_{i-1}}{\Delta t}, \tag{1}$$

$$e(t) = V - V_0, \tag{2}$$

$$u(t) = K_p e(t) + \frac{1}{T_i}\int_0^t e(t)dt + T_d \frac{de(t)}{d(t)}, \tag{3}$$

where $e(t)$ is the error value obtained by subtracting $V - V_0$, and $u(t)$ is the control function of the pump. Moreover, K_p , T_i and T_d terms are the proportional coefficient, the integral time constant, and the differential time constant, respectively.

3. The Inflation-Type BP Measurement

Regard with cuff-based BP measurement methods, it is required to have around 30~40 mmHg inflation gap between the maximum inflation pressure and the real SBP situation for a person with 80 heartbeat rate. For example, if a person’s SBP is 120 mmHg, the cuff device must be pumped to 150~160 mmHg. Otherwise, its measurement may involve noise (over 160 mmHg) or lose accuracy (under 150

mmHg). This inflation gap size may be different for different people. Results in Figure 3 show that our device can be automatically pumped to the appropriate maximum inflation pressure values according to the preset systolic blood pressure. Additionally, to provide more personalized measurement sets for different stroke patients, this device can be adapt to physiological and external factors as well.

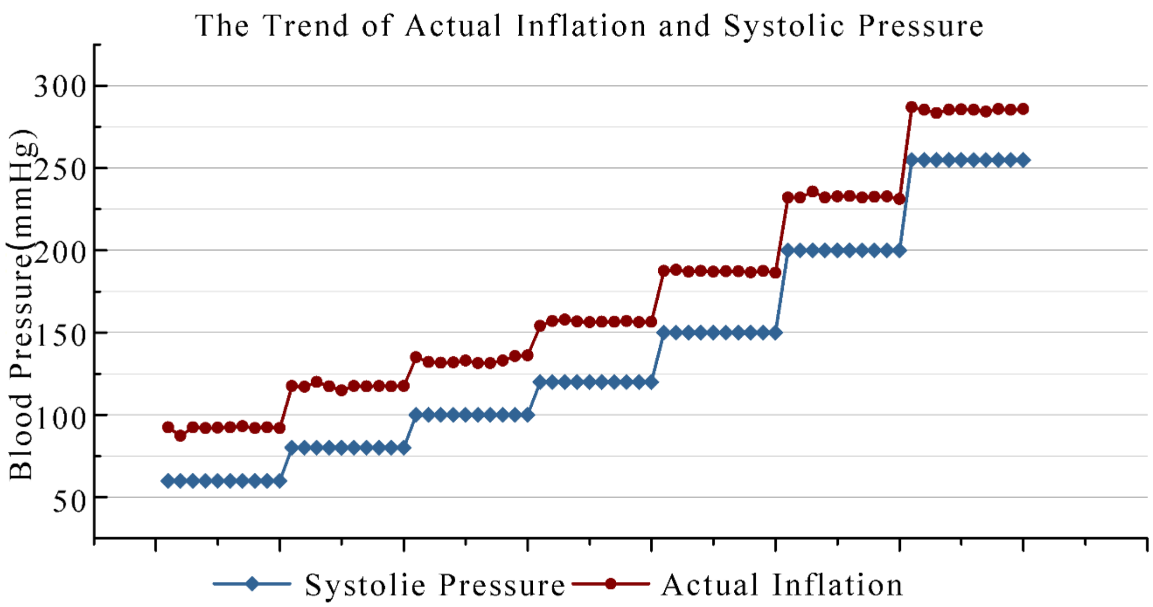

Figure 3. The maximum inflation pressure value be self-adaptive to its corresponding systolic pressure.

4. Device test using simulated signals

Table 1. Results of our device tests.

BP Types	Preset BP Values		Measurement Values of Our Device with Algorithm 1										Omron HEM-7 207
Standard BP I	SP	60	60.90	60.87	60.91	60.71	60.97	60.89	60.99	60.91	60.83	60.87	Out of Range
	DP	30	30.60	30.46	30.61	30.53	30.55	30.57	30.58	30.38	30.37	30.47	Out of Range
Standard BP II	SP	80	80.38	79.92	79.92	80.44	79.52	79.32	79.26	80.37	80.24	80.29	80
	DP	50	50.37	50.70	50.96	50.19	49.62	49.85	49.33	50.33	49.97	50.48	49
Standard BP	SP	100	100.67	100.32	100.81	100.81	100.70	100.55	101.19	101.04	100.80	100.75	99

III	DP	65	64.95	65.21	65.54	65.15	65.25	64.99	64.97	64.84	65.10	65.22	65
Standard BP	SP	120	119.36	119.89	120.49	120.57	120.12	119.85	120.14	120.32	121.07	120.58	118
IV	DP	80	80.42	80.25	80.74	80.59	80.72	80.64	80.47	80.41	80.42	80.43	81
Standard BP	SP	150	150.58	151.08	150.96	150.78	150.63	150.35	150.07	150.25	151.07	151.26	147
V	DP	100	100.81	100.72	100.87	100.59	100.14	100.08	100.47	100.55	100.38	99.95	101
Standard BP	SP	200	199.51	199.89	199.86	199.89	199.53	199.63	199.67	199.45	200.14	200.19	196
VI	DP	150	149.95	149.89	150.13	149.65	150.10	149.99	149.76	149.79	149.40	149.34	152
Standard BP	SP	255	254.44	253.90	253.47	254.32	254.34	253.79	254.92	254.16	254.88	253.57	Out of Range
VII	DP	195	194.62	194.41	194.44	194.39	194.20	194.26	194.62	194.87	194.24	194.71	Out of Range
Weak Pulse	SP	110	109.61	109.31	109.46	109.60	109.60	109.60	109.60	109.89	109.61	109.61	109
	DP	80	79.63	79.60	79.72	79.36	79.36	79.36	79.36	79.12	79.20	79.20	81
Mild Exercise	SP	140	139.63	139.36	139.57	139.52	139.93	139.67	139.89	140.16	144.78	139.81	141
	DP	90	89.06	89.60	89.14	89.21	89.06	89.48	89.22	89.10	89.07	89.21	90
Strenuous	SP	140	140.24	140.38	140.95	141.00	140.91	141.11	141.03	141.03	140.68	141.12	143
Exercise	DP	90	89.24	89.22	88.64	88.88	88.53	88.48	88.79	88.72	88.87	88.70	91
Fat	SP	120	119.86	120.02	119.36	119.57	119.35	120.09	119.39	119.78	119.78	119.55	118
	DP	80	80.69	80.47	80.82	80.48	80.54	80.64	80.67	80.64	80.79	81.04	82
Elderly	SP	150	150.29	150.73	150.63	150.09	150.76	150.79	150.07	150.40	150.23	150.76	147
	DP	110	109.80	109.40	108.32	108.63	108.15	108.73	108.82	109.73	109.34	109.48	113
Tachycardia	SP	120	118.94	118.85	119.11	118.61	119.12	119.00	118.58	118.62	118.48	118.66	122
	DP	105	103.08	103.01	102.87	103.45	102.67	103.42	103.80	103.28	102.82	103.74	105
Bradycardia	SP	120	123.97	123.88	123.84	124.07	123.87	124.02	123.84	123.86	123.83	124.01	122
	DP	60	59.46	59.76	59.62	59.46	58.71	58.73	58.88	58.75	58.88	58.97	59
Arrhythmias I	SP	138	139.59	139.94	137.68	136.88	138.80	138.19	139.68	138.22	138.67	136.96	131
	DP	53	56.05	56.37	56.97	56.87	57.69	57.91	56.74	57.05	58.18	58.02	57
Arrhythmias	SP	144	140.73	140.97	140.76	143.71	142.95	142.78	144.67	143.03	143.12	144.99	139
II	DP	64	69.68	69.85	70.13	66.10	67.90	67.43	67.43	68.27	67.90	66.51	67
Arrhythmias	SP	118	120.38	119.60	120.40	120.90	120.37	120.37	119.45	120.74	120.97	120.70	111
II	DP	61	58.33	58.11	59.39	58.84	58.66	58.66	58.12	59.02	58.62	59.55	59
Respiratory	SP	138	137.96	137.86	138.30	138.08	138.62	138.46	138.50	138.16	138.35	138.05	132
Interference I	DP	65	68.43	68.79	68.05	68.04	67.70	67.90	68.15	68.24	68.43	68.36	70
Respiratory	SP	149	144.28	145.16	145.70	144.55	144.70	144.57	145.38	144.48	145.53	145.55	145

Interference II	DP	65	68.18	68.81	68.05	68.59	68.87	67.96	67.13	68.52	67.08	66.98	66
Respiratory Interference III	SP	112	112.46	113.27	112.14	112.80	112.80	112.76	112.63	112.03	111.90	112.72	106
	DP	47	48.83	48.47	49.12	48.43	48.80	48.50	48.55	48.20	48.91	48.42	52
