# Supplementary material for: An Ambulatory Blood Pressure Monitor Mobile Health System for Early Warning for Stroke Risk: Longitudinal Observational Study
Source: JMIR Mhealth Uhealth. 2019 Oct 30;7(10):e14926. doi: 10.2196/14926 (PMC6913731; doi:10.2196/14926)
Supplement: Multimedia Appendix 2 [file mhealth_v7i10e14926_app2.pdf]

# Multimedia Appendix 2

## An Abnormal BP data Analyzing Algorithm for Stroke-Risk Early Warning

In this ABA algorithm, the adopted SVM strategy can map samples to a high-dimensional feature space nonlinearly and find the optimal separating hyperplane *y=<w, x>+b*, which is described by the following quadratic programming optimization problem:

$$\min \frac{1}{2}\|w\|^2 + C\sum_{i=1}^{n}\xi_i \qquad (4)$$

$$s.t.\{ \begin{matrix} y_i[<w, x_i> + b] \geq 1 + \xi_i \\ \xi_i \geq 0 \end{matrix} ,$$

where $\xi_i$ is the relaxation factor, *C* is the penalty coefficient, and Radial Basis Function (RBF) is chosen as the kernel function. To improve performance, Simulated Annealing (SA) is used to tune the SVM hyper-parameters, such as *C* and RBF parameter *g*. In our experiment, 60%, 20%, and 20% of data are used as training, validation, and test set, respectively. Additionally, the 10-fold cross-validation is applied for refining hyper-parameters. The ABA algorithm is shown in Algorithm 2.

**Algorithm 2:** Abnormal BP Data Analyzing (ABA) Algorithm.

Objective Function:

$f_{of}$ (which is the error rate of stroke risk early prediction under the constraint of suitable SVM parameters $P_0 = [g, C]$, which are optimized by the SA computing strategy.)

Input:

Initial temperature: $T_0$

ending temperature: e

decay scale: η

cycle index: *M*

patients' data sets and features: *X*

Randomly generate $P_0$, Set $P_{opt} = P_0$, $f_{opt} = f_{of}(P_0)$.

**for** *m* = 1 to *M* **do**

randomly generate $P_{i+1} = P_i + \eta^T \gamma$ , while $\gamma$ is a random perturbation vector which follows Cauchy distribution.

Calculate objective function $f_{of}(P_{i+1})$ and increment $\Delta t = f_{of}(P_{i+1}) - f_{of}(P_i)$ . // this substep is to improve the precision level of a stroke high-risk event prediction.

**If** $\Delta t \le 0$ **then**

$P_{opt} = P_{i+1}$ , $f_{opt} = f_{of}(P_{i+1})$

**else**

determine whether or not to accept the new values by Metropolis rule. Randomly generate $\mu \in [0,1]$,

**if** $\mu < \exp(-\frac{\Delta t}{T})$ **then**

$P_{opt} = P_{i+1}$ , $f_{opt} = f_{of}(P_{i+1})$,

**else**

stay the same.

**end if**

**end if**

**if** $T < e$ **then**

break.

**end if**

**end for**

Use the final optimized hyper-parameters $P_{opt}$ and data sets *X* to train SVM model.

//This step is to perform stroke early risk warning prediction with precision.
Use this model to predict users' stroke risk levels.
**Output:** Stroke risk early warning.

---
